# Integrated HIV care and service engagement among people living with HIV who use drugs in a setting with a community-wide treatment as prevention initiative: a qualitative study in Vancouver, Canada

**DOI:** 10.7448/IAS.20.1.21407

**Published:** 2017-03-03

**Authors:** Alexandra B Collins, Surita Parashar, Robert S Hogg, Saranee Fernando, Catherine Worthington, Patrick McDougall, Rosalind Baltzer Turje, Ryan McNeil

**Affiliations:** ^a^ BC Centre for Excellence in HIV/AIDS, Vancouver, BC, Canada; ^b^ Faculty of Health Sciences, Simon Fraser University, Burnaby, BC, Canada; ^c^ School of Public Health and Social Policy, University of Victoria, Victoria, BC, Canada; ^d^ Dr. Peter AIDS Foundation, Vancouver, BC, Canada; ^e^ Department of Medicine, University of British Columbia, Vancouver, BC, Canada; ^f^ British Columbia Centre on Substance Use, Vancouver, BC, Canada

**Keywords:** HIV/AIDS, integrated service model, treatment as prevention, structural vulnerability, qualitative, Canada

## Abstract

**Introduction**: Social-structural inequities impede access to, and retention in, HIV care among structurally vulnerable people living with HIV (PLHIV) who use drugs. The resulting disparities in HIV-related outcomes among PLHIV who use drugs pose barriers to the optimization of HIV treatment as prevention (TasP) initiatives. We undertook this study to examine engagement with, and impacts of, an integrated HIV care services model tailored to the needs of PLHIV who use drugs in Vancouver, Canada – a setting with a community-wide TasP initiative.

**Methods**: We conducted qualitative interviews with 30 PLHIV who use drugs recruited from the Dr. Peter Centre, an HIV care facility operating under an integrated services model and harm reduction approach. We employed novel analytical techniques to analyse participants’ service trajectories within this facility to understand how this HIV service environment influences access to, and retention in, HIV care among structurally vulnerable PLHIV who use drugs.

**Results**: Our findings demonstrate that participants’ structural vulnerability shaped their engagement with the HIV care facility that provided access to resources that facilitated retention in HIV care and antiretroviral treatment adherence. Additionally, the integrated service environment helped reduce burdens associated with living in extreme poverty by meeting participants’ subsistence (e.g. food, shelter) needs. Moreover, access to multiple supports created a structured environment in which participants could develop routine service use patterns and have prolonged engagement with supportive care services. Our findings demonstrate that low-barrier service models can mitigate social and structural barriers to HIV care and complement TasP initiatives for PLHIV who use drugs.

**Conclusions**: These findings highlight the critical role of integrated service models in promoting access to health and support services for structurally vulnerable PLHIV. Complementing structural interventions with integrated service models that are tailored to the needs of structurally vulnerable PLHIV who use drugs will be pursuant to the goals of TasP.

## Introduction

The negative impact of social-structural inequities, such as poverty, homelessness and drug criminalization on HIV treatment and care among people living with HIV (PLHIV) who use drugs is well documented [1–4]. The marginal position that drug-using populations occupy within social hierarchies due to the intersection of political (e.g. drug criminalization), economic (e.g. poverty), and sociocultural (e.g. anti-drug stigma, racism) arrangements renders them what has been termed “structurally vulnerable” [5,6] to adverse HIV-related outcomes [7]. In any given context, dynamics within the HIV risk environment – the settings or situations in which micro- and macro-level forces (i.e. physical, social, economic and policy conditions) intersect to shape HIV-related outcomes [8,9] – frame the structural vulnerability of PLHIV who use drugs and can undermine their access to highly active antiretroviral therapy (HAART) and retention in HIV care [9–11].

Addressing social-structural inequities that negatively impact drug-using populations’ access to HIV treatment and retention in HIV care is particularly urgent in the context of HIV Treatment as Prevention (TasP) [12,13]. TasP aims to produce viral suppression among PLHIV by expanding access and improving adherence to HAART and retention in HIV care to prevent the onward transmission of HIV, and achieve reductions in HIV-related morbidity and mortality [14]. As the basis of the UNAIDS 90–90–90 campaign [15], TasP holds particular promise in extending the preventive and life-saving qualities of HAART to structurally vulnerable drug-using populations [12,16] who have historically benefited less than other PLHIV from HIV treatment advances [17–19]. Understanding how dynamics within the risk environments of PLHIV who use drugs shape access and engagement with HIV care in the context of TasP initiatives will help to inform the optimization of this intervention.

Previous research has identified social-structural influences, such as stigma [20,21], lack of social supports [3,20,22,23], and unstable housing [24–27] on access to HIV-related care, particularly for PLHIV who use drugs. However, considerably less attention has been paid to how HIV care services designed to be responsive to the structural vulnerabilities of drug-using populations can shape engagement and retention in HIV care. Integrated service models, which provide multiple services that address co-occurring needs (e.g. HIV, mental health, hepatitis C) within one facility [28,29], have demonstrated potential to mitigate the effects of individual structural vulnerabilities on HIV care by creating a person-centred continuum of care tailored to the needs of specific populations. To date, the limited research on integrated HIV care services has primarily focused on clinical outcomes [30–34], highlighting their potential to improve HAART-related outcomes [10, 35–37]. However, as Christopoulos and colleagues note, competing priorities (e.g. comorbid conditions, substance use) often take precedence over HIV care management for PLHIV who use drugs, even when integrated services are available [30]. This literature suggests an urgent need to further tailor integrated service models to account for the social-structural forces operating within the risk environments of structurally vulnerable PLHIV who use drugs. Of particular importance will be understanding how drug-using populations engage with integrated HIV care services, including the specific services that facilitate their engagement. In settings implementing TasP, such engagement will be necessary to achieve population-level targets for retention in HIV care.

This is especially relevant in Vancouver, British Columbia, Canada, home to a large HIV-positive drug-using population [14,31]. Vancouver became among the first jurisdictions to implement TasP [32] through an initiative that aimed to expand testing to identify PLHIV, provide them with no-cost HAART, and link them to HIV care. The Dr. Peter Centre (DPC), a Vancouver-based HIV care service organization, employs an integrated services model and provides services to approximately 425 PLHIV annually. The DPC aims to improve access to, and retention in, HIV care by providing services responsive to forces operating within the HIV risk environment that adversely impact outcomes among structurally vulnerable PLHIV, including HAART support (see overview in Table 1). Since 2002, the DPC has also integrated comprehensive harm reduction (HR) services (including nurse-supervised injection services) to minimize drug-related harm and address the diverse needs of clients [10,33]. The DPC thus provides a unique context in which to explore integrated service models within the context of an evolving HIV epidemic and following the implementation of a TasP initiative. We undertook this study to generate insights into how the DPC’s integrated services model influences access to, and retention in, HIV care among structurally vulnerable PLHIV who use drugs, and to understand the onward impact on HIV-related outcomes.

**Table 1.** DPC integrated services model.

Risk environment	Dr. Peter Centre Services
Physical	
• Unsafe injection associated with injection in public spaces and shooting galleries [42,43]	• Integrating supervised injection services into the DPC residence and day health programme
• Homelessness and housing instability associated with lack of opportunities for self-care [26]	• Day health programme provides access to services meeting basic needs (e.g. showers, sleep rooms, laundry facilities, food services)
• Discharge from hospital against medical advice associated with injection drug use and abstinence-only policies in health settings [44,45]	• DPC residence provides supervised injection services to minimize disruptions in care that occur due to continued injection drug use
Social	
• Experiences of drug-related stigma in interactions with health care professionals [44,45]	• Mandatory harm reduction (HR) training for staff combined with comprehensive, low-threshold nursing care services (e.g. health assessments, medication assistance, HAART support, symptom management)
• PLHIV who use drugs experiencing high levels of violence and interpersonal conflict [44,46]	• Enabling the development of positive relationships by providing social support and programmes (e.g. recreational therapy outings, karaoke)
• High levels of depression, severe mental illness, and suicide among persons who inject drugs [47,48]	• Residence and day health programme provide counselling services, including art and music therapies, and mental health resource referrals
Economic	
• High levels of food insecurity and hunger among PLHIV, particularly women, Indigenous persons, and people who inject drugs [49,50]	• Day health programme provides nutrient-dense meals twice daily, seven days per week, and residence provides clients with regular meals and snacks
• Housing instability and homelessness associated with poor overall health and increased mortality [12,27]	• Staff provide referrals to supportive and subsidized housing, particularly housing intended for PLHIV
• High unemployment and limited economic opportunities associated with involvement in illegal and informal income generation [51,52]	• Staff provide assistance filling out paperwork for social welfare entitlements
Policy	
• Reluctance or difficulty accessing clinical and support services (primary, respite, and end-of-life care services) due to abstinence-only drug policies and drug criminalization [44,53]	• Adopts comprehensive HR model to minimize barriers PLHIV who inject drugs face when accessing care services and consults with local decision-makers (e.g. policymakers, police) to increase awareness of the public health benefits of this approach
	• Professional staffing model and best practices in culturally sensitive programming employed to create safer environments for clients

Dr. Peter Services that address aspects of clients’risk environments at physical, social, economic and policy levels.

## Methods

We draw upon 30 semi-structured, qualitative interviews conducted with PLHIV who use drugs and are clients of the DPC. The interviews are part of a qualitative sub-study of a community-based research project exploring engagement with the DPC among structurally vulnerable PLHIV. As noted elsewhere [34], this mixed-methods study includes: a longitudinal cohort of recently enrolled DPC clients who complete baseline (*n* = 121) and follow-up (*n* = 102) sociobehavioural surveys; and qualitative interviews (*n* = 30) (see Table 2).

**Table 2.** Participant characteristics.

Participant characteristic	*n* (%) *n = 30*
Age	
Mean	46.6
Range	26–77 years
Gender	
Men	24 (80.0%)
Women	4 (13.3%)
Transgender	2 (6.66%)
Race	
White	19 (63.3%)
Indigenous ancestry	8 (26.7%)
Other	3 (10.0%)
Sexual orientation	
Straight	14 (46.7%)
Gay	13 (43.3%)
Bisexual	3 (10.0%)
Current housing	
Single room occupancy hotel	6 (19.3%)
Apartment	15 (51.6%)
Unsheltered	0 (0.00%)
Other^a^	9 (29.0%)
Substance use^b^	
(30 days prior to interview)	
Other opiates (including methadone)	20 (31.7%)
Crystal methamphetamine	18 (28.6%)
Heroin	10 (15.9%)
Crack cocaine	9 (14.3%)
Powdered cocaine	6 (9.52%)
HAART adherence^c^	
(12 months prior to qualitative interview)	
76–100% HAART adherence	5 (16.7%)
51–75% HAART adherence	13 (43.3%)
26–50% HAART adherence	6 (20.0%)
0–25% HAART adherence	6 (20.0%)

^a^Includes: social housing; basement suite; emergency shelter; and house.

^b^Drug categories are not mutually exclusive.

**^c^**According to validated pharmacy refill measure obtained through external data linkage (HAART adherence % = (HAART_days/365) ˟ 100).

Qualitative interview participants were recruited from longitudinal cohort participants who had completed baseline surveys as of September 2014. Of the 98 longitudinal cohort participants who had completed baseline surveys at that time, 85 (87%) reported illicit drug use (excluding marijuana) in the previous six months and provided written consent to be contacted regarding participation in qualitative interviews. A database query of cohort data for these 85 individuals was conducted to obtain demographic characteristics and HIV-related clinical outcomes (e.g. HAART adherence). We aimed to oversample women and Indigenous persons relative to their representation among DPC clients, and to recruit participants with varying levels of HAART adherence. Information letters were sent in waves to eligible participants to allow for purposeful sampling. The first wave prioritized women and people of Indigenous ancestry, with subsequent waves prioritizing participants of varying sexual orientations and levels of HAART adherence.

Interviews were conducted in a private meeting room at the DPC between December 2014 and April 2015 by three trained interviewers. The interviewers explained the study to participants, answered any questions, and obtained written informed consent. An information form was used to collect demographic information (e.g. drug use patterns, housing status) and an interview guide was used to facilitate discussion regarding participants’ perspectives on DPC services and factors impacting DPC service utilization. Interviews were approximately 60 min in length and audio recorded. Participants received $30 CAD honoraria as compensation for their time. A professional transcription service was used to transcribe interviews, which were later reviewed for accuracy by the interviewers. Ethical approval was obtained from the Providence Healthcare/University of British Columbia and Simon Fraser University research ethics boards.

Interview transcripts were imported into NVivo qualitative analysis software to facilitate coding and thematic extraction using inductive methods [35]. The research team met regularly to discuss emerging themes and the application of theory to the interpretation of findings. For this analysis, the lead author mapped out participants’ trajectories and points of engagement with DPC services based on interview data, and characterized how participants navigated the DPC’s integrated services model. Interview transcripts were reviewed to create preliminary DPC service trajectories for each participant. Service utilization was documented sequentially in list form, then organized based on point of entry – the first use of a DPC service, including first service accessed following an extended break (i.e. six months or more). Members of the research team reviewed preliminary trajectories to discuss potential themes. Flow diagrams were then constructed beginning with participants’ point of entry and followed participants’ DPC service engagement. Multiple points of entry were noted, with contextual information relating to points of entry (e.g. housing status, drug use) added to facilitate analysis.

**Figure 1.**
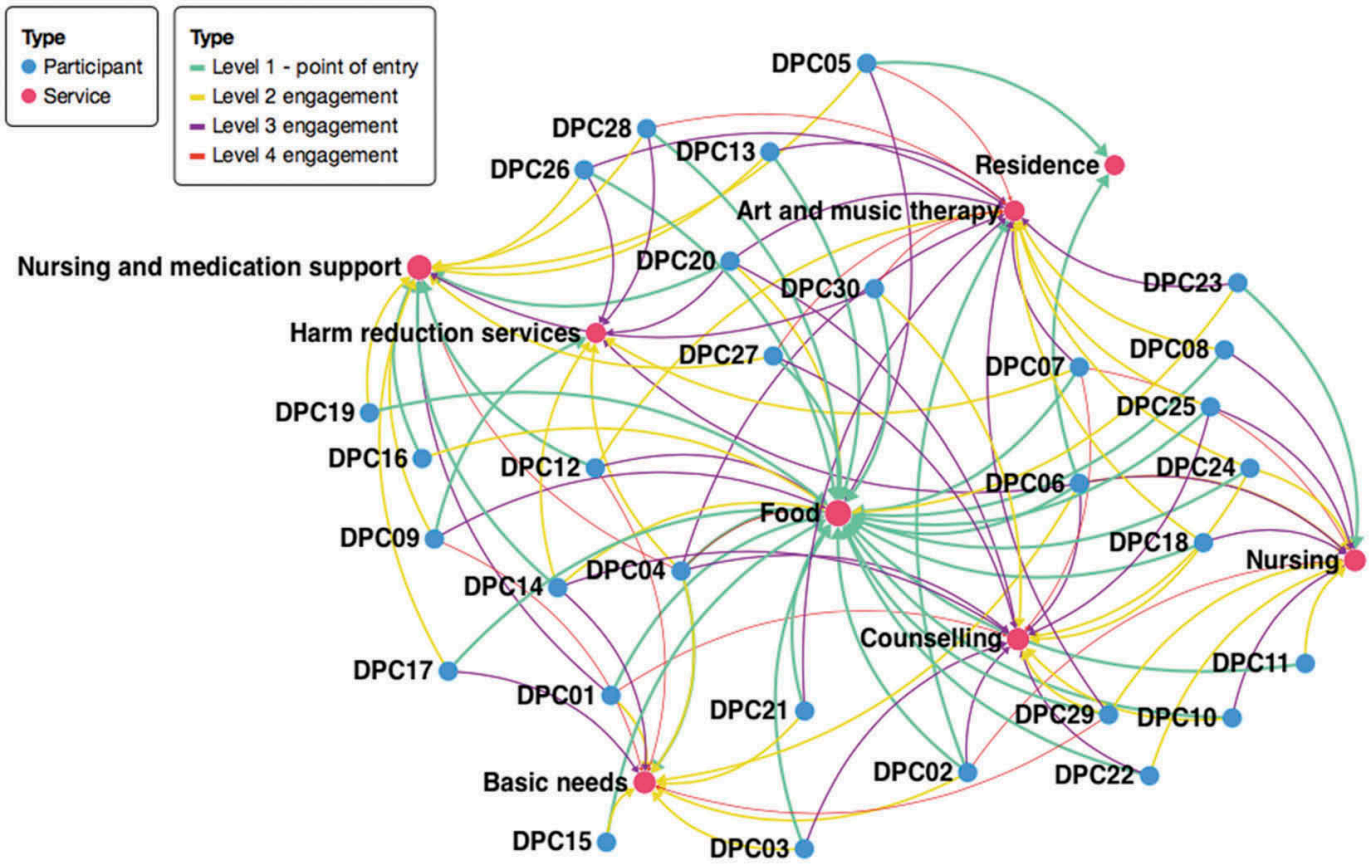
Aggregate participant trajectories by levels of engagement (*n* = 30). Coloured lines represent levels of engagement with the integrated service environment (teal: point of entry; yellow: second level engagement; purple: third level engagement; red: fourth level engagement).

Each diagram was then analysed for themes between participants’ points of entry and structural vulnerability. Analysis was conducted using risk environment and structural vulnerability frameworks [6,8], and organized based on point of entry. Diagrams were uploaded into Microsoft PowerPoint software to allow for easy reorganization based on emergent themes. An aggregate service pattern was mapped based on cumulative data from participants’ individual trajectories (see Figure 1). Members of the research team met to discuss emerging patterns and interpretations. Trajectories were then reanalysed using Polinode, a social network analysis software, following the establishment of the final themes to ensure their credibility.

## Findings

### Points of entry to an integrated HIV care environment

Participants’ structural vulnerability shaped their initial engagement with the DPC’s integrated services model, with their point of entry reflecting their most urgent need at that particular time. Because the DPC’s intake procedure prioritizes clients with complex health and social care needs (e.g. housing instability, complex comorbidities), participants had access to an integrated services model tailored to their structural vulnerabilities upon intake. Depicting aggregate service pattern data, Figure 1 illustrates how the initial services accessed by many participants were directly related to their structural vulnerabilities (e.g. food insecurity), while subsequent services reflected a greater range of health and social care needs (e.g. counselling, basic needs (i.e. shower, laundry facilities, nap room)). For most participants (*n* = 23), nutrient-dense meals provided through the DPC’s food service were the first accessed service, as well as the most commonly accessed service by all participants. Other subsistence needs were also commonly represented among the first services accessed by participants (nursing (*n* = 1), nursing and HAART support (n = 4), basic needs (n = 1) and residence (n = 2)), underscoring their importance within this integrated HIV services model (see Figure 1).

Nursing and HAART adherence support were the second most frequent points of entry, with approximately half of participants (*n* = 14) reporting having their HAART dispensed at the DPC. However, some participants accessed only nursing services (e.g. medical assessments, first aid), having their medication dispensed elsewhere. While these participants were typically better resourced at the time of enrolment in the DPC (e.g. stably housed), they emphasized that social-structural forces, such as abstinence-based policies, operating within the risk environment in health settings nonetheless impeded their access to services, including HIV treatment and care. For example, experiences of being “treated like a criminal” or “like an inconvenience” by nurses and physicians due to drug use created barriers to retention in care. Among these participants, access to a low-threshold integrated HIV care model, including HAART adherence supports and other ancillary supports (e.g. support groups, arts-based therapy), in which they were not stigmatized based on their drug use, was crucial to fostering overall engagement in HIV care.

### Engaging with an integrated service environment

**Figure 2.**
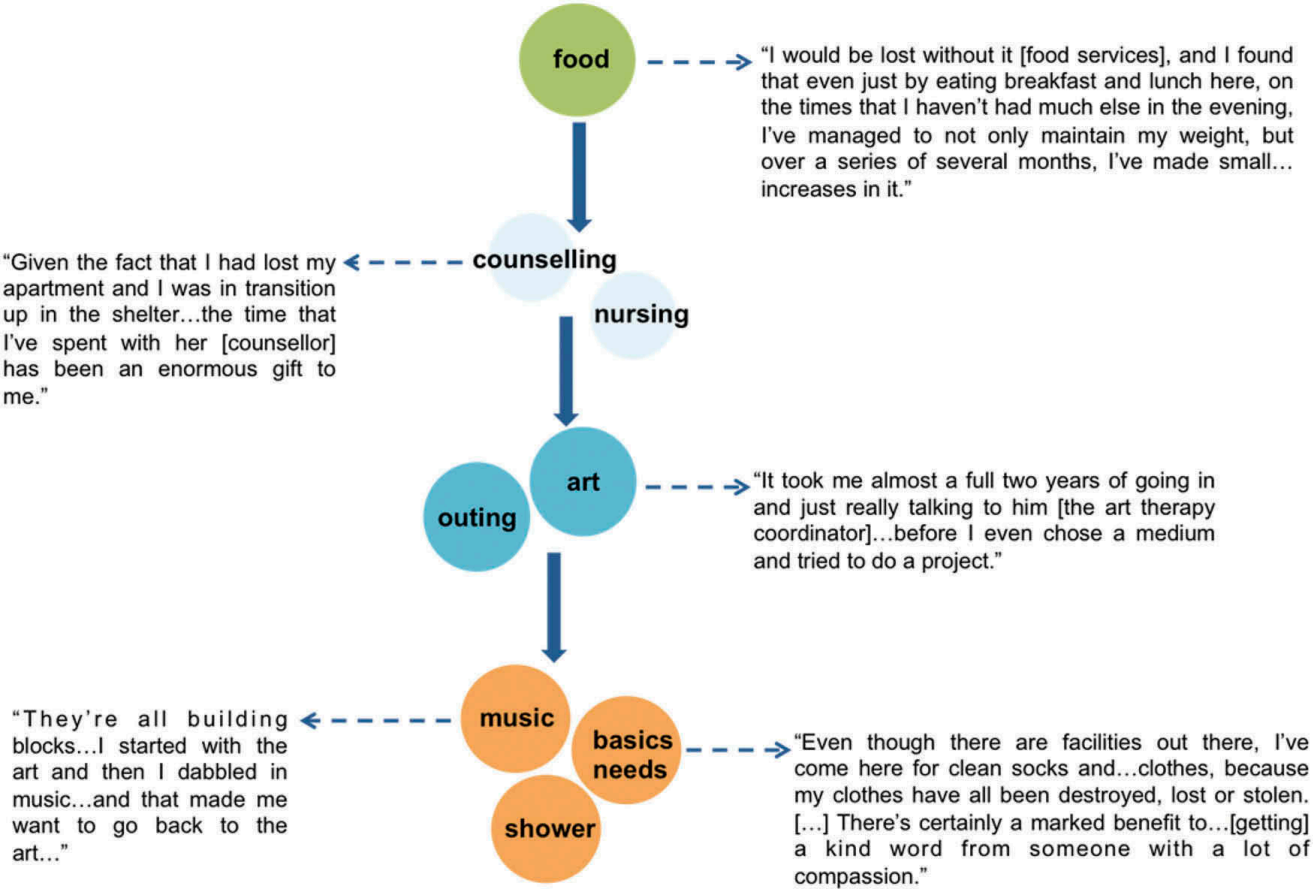
Accessing multiple services to meet varying needs (49-year-old White man). Participant’s trajectory accessing DPC services, starting with point of entry.

Participants’ types of DPC engagement were not limited to one specific service, and evolved over time in response to their structural vulnerabilities. Through their initial engagement with the DPC, participants gained access to a service environment with programmes tailored to their needs (e.g. nutrient-dense meals, housing, HIV care), and their subsequent engagement across these services enabled them to attenuate structural vulnerabilities. For example, Figure 2 depicts a service trajectory common among participants and demonstrates how engagement within this integrated service environment was shaped by structural vulnerability. Although nutrient-dense meals served as the entry point to DPC services, this participant accessed counselling and housing supports to address traumatic experiences and challenges relating to housing instability. In addressing these structural vulnerabilities, this participant then proceeded to access DPC services (e.g. arts therapy) that addressed additional complex challenges, such as social isolation and low self-worth.

Participant narratives demonstrated that the DPC’s tailored and multifaceted services reduced the hardships associated with living in extreme poverty while better accommodating competing demands (e.g. medical appointments, HR services). Although some participants quickly began accessing an array of services upon enrolling at the DPC, others articulated the need to develop “comfort” with the DPC’s programme environment and staff over time. In the latter situation, specific programmes (e.g. drop-in services, support groups) that fostered community among DPC clients and trust with DPC staff facilitated engagement with new programmes. For example, one participant described how she was encouraged to access new services based on suggestions of support group members:

I didn’t want people to know [I had HIV] because I hadn’t disclosed to my family …I [came] just when I needed to for food and then I started realizing bit by bit how much they really had here […] The women’s group is really great … we can talk about our issues and stuff […] You can find that you have [stuff] in common and the ways to get out of your ruts … it got me into checking out the art room. (44 year-old, Indigenous woman)

Similarly, participants reporting a greater degree of social integration at the DPC (e.g. positive peer and staff relationships) accessed a wider variety of services, including arts therapy, counselling, and HR services.

### Improving HIV care through integrated services

Participants reported that, while social-structural forces, such as stigma in health care encounters and extreme poverty, negatively influenced HIV disease management, treatment supports (e.g. HAART dispensation, in-person reminders) provided as part of the DPC’s service model enabled them to maintain HAART adherence and retention in HIV care. Importantly, because support services designed to address survival needs (e.g. food services) were co-located with HAART adherence supports and HIV care, participants believe that the DPC’s overall integrated services model fostered improvements in perceived HIV-related outcomes. For example, as one participant explained, having all their needed services “under one roof” ensured that participants’ HIV care plans and support services were coordinated:

I switched everything under one roof. […] [In the past] he [my doctor] made a few big mistakes, he made some serious mistakes…[At the DPC] they always make sure, by keeping things under one roof, that all the meds I’m taking combine and go with the treatments I have … That’s why I kept things between the DPC. (50 year-old, White man)

For other participants, the geographic location of the DPC was identified as facilitating regular engagement in HIV care, as it was located outside of the service-rich neighbourhood that is home to a prominent drug scene that participants reported “triggered” their drug use.

Additionally, participants indicated that various DPC services enabled them to better manage mental health challenges and other aspects of their lives, including medication adherence and ongoing care for non-HIV-related conditions. For example, one participant expressed:

I’m happy now. I don’t get depressed to the point where I feel like going into something and using. It was a negative thing and now I’m managing everything about my life better with these supports in place. (44 year-old, Indigenous woman)

As such, the DPC service model not only addressed participants’ immediate needs relative to their structural vulnerability, but also helped participants better manage their health and wellbeing.

### Routine service patterns and improvements in HIV treatment and care

**Figure 3.**
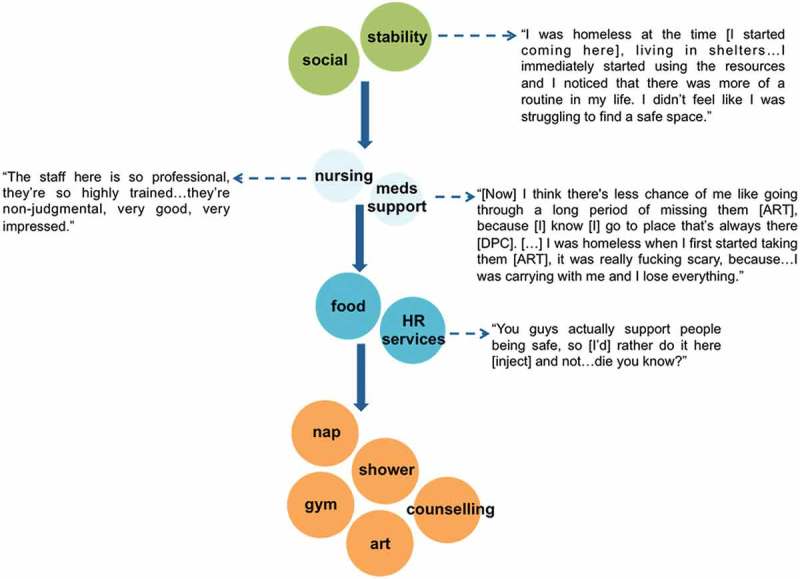
Routines and safe spaces (29-year-old White man). Participant’s trajectory accessing DPC services, starting with point of entry.

Participant accounts suggest that, because this integrated services model was tailored to the needs of structurally vulnerable PLHIV who use drugs, it led to prolonged and routine service engagement and enabled participants to meet basic needs that were not – or could not – be met otherwise. Participant narratives demonstrated that the DPC service model fostered access to various supports and services that were essential to survival and HIV disease management. For many participants, access to nutrient-dense meals, HAART adherence supports and culturally safe nursing care fostered routine service engagement and was reported as contributing to improvements in HIV-related outcomes and overall wellbeing. Notably, this comprehensiveness of programming was particularly responsive to any disruptions experienced by participants that commonly pose barriers to care, including periods of homelessness and acute mental health challenges. As outlined in Figure 3, the DPC’s integrated services model provided both stability and structure when a participant experienced disruptions to their daily life, and enabled them to better manage their HIV. For this participant, the onsite dispensation of HAART and low-threshold service environment served as critical environmental supports that enabled HAART adherence and retention in HIV care when experiencing homelessness.

Importantly, our analysis indicated that the DPC’s programming led to prolonged engagement during the organization’s operating hours, thereby supporting the development of daily routines. As one participant noted:

It’s very easy: you come here, eat breakfast, you see a nurse, you do art therapy, you wait for the next class, you drink tea, you sit in front of the fire, you know computers, everything. It’s really comfortable. […] It’s perfect. (48 year-old White woman)

**Figure 4.**
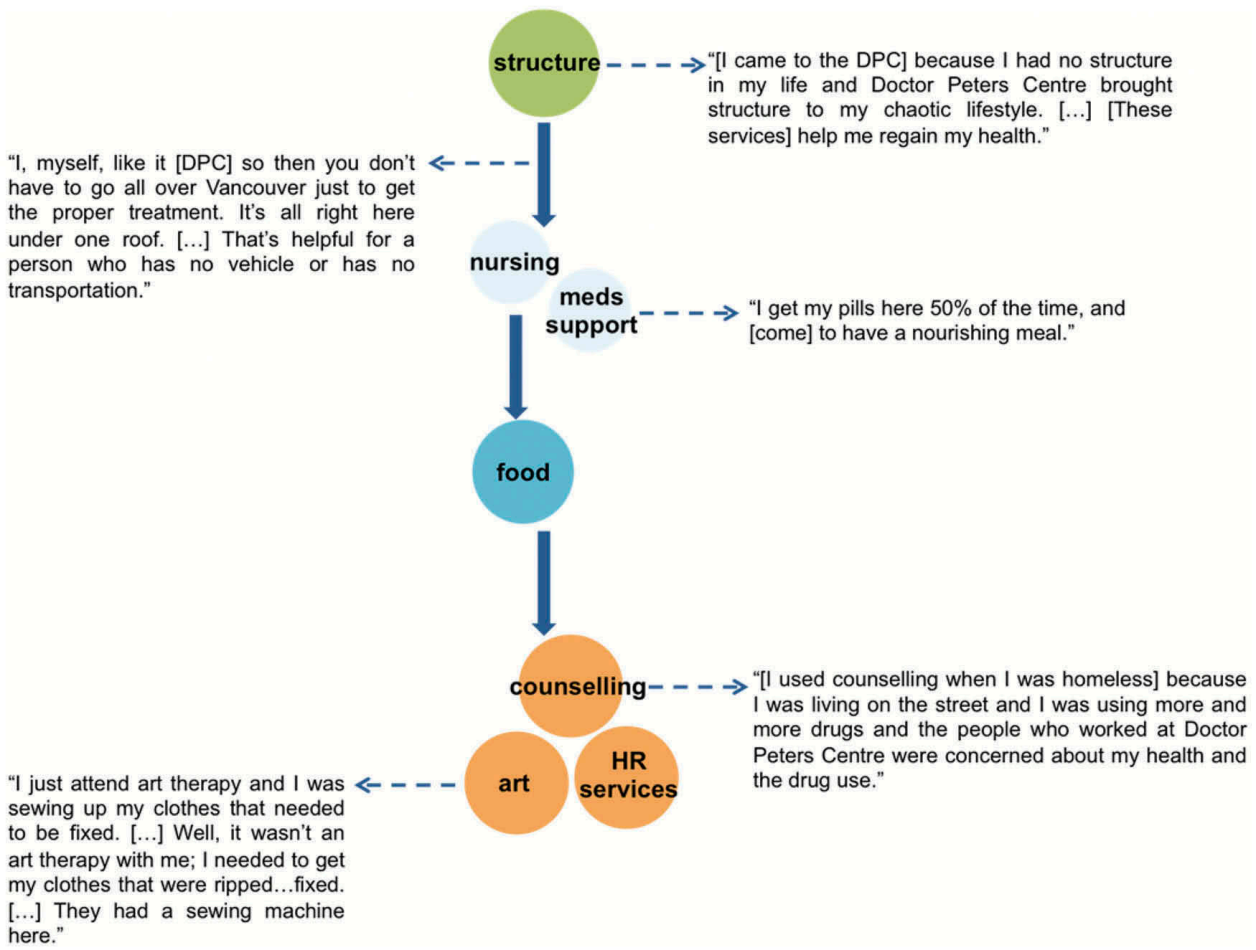
Finding structure in one place (50-year-old Indigenous man). Participant’s trajectory accessing DPC services, starting with point of entry.

Among participants, access to multiple services at the DPC was commonly characterized as comprehensive because the availability of multiple services eased the requirements necessary to achieve retention in care. As outlined in Figure 4, one participant explained how the DPC service model allowed him to better manage his health by providing a structured environment he could continuously engage in. In doing so, the DPC not only alleviated some challenges stemming from the participant’s structural vulnerability (e.g. insufficient resources for transportation), but also provided supports critical to fostering a stable environment conducive to HAART adherence and retention in HIV care.

## Discussion

In summary, we found that the DPC’s integrated services model engages PLHIV who use drugs by responding to their structural vulnerabilities, which participants reported improved their HAART adherence and retention in HIV care. Although programming responsive to specific structural vulnerabilities (e.g. food insecurity) was an initial point of entry into DPC, service engagement evolved over time as specific structural vulnerabilities were addressed (e.g. housing instability, meeting basic needs), and as participants accessed services to attend to other needs. Routine engagement with the DPC’s integrated services model functioned to minimize structural barriers to health, creating regular engagement with services and HIV-related care. Overall, our findings demonstrate how a low-barrier, integrated services model can mitigate social-structural barriers to retention in HIV care and support TasP initiatives for structurally vulnerable populations who use drugs.

Previous research has demonstrated the importance of complementing TasP with structural interventions to improve access to, and engagement with, HIV treatment and care [3,7,11,12,33,36]. Our findings add to this literature by demonstrating the importance of further complementing structural interventions – in this case, TasP – with integrated service models tailored to the needs of structurally vulnerable PLHIV who use drugs. By incorporating programming responsive to barriers to engagement with HIV treatment and care (e.g. HR programming, food services) in a setting with a community-wide TasP initiative into its integrated services model, the DPC represents one such targeted approach that demonstrates promise in optimizing HIV-related outcomes among PLHIV who use drugs. Such programming may be beneficial to optimize TasP among drug-using populations in other settings by facilitating access to, and retention in, HIV care. While the DPC service model addressed various dynamics within the risk environment, social-structural forces (e.g. drug-related stigma, neighbourhood deprivation, social dynamics within the street-based drug scene) continued to pose challenges to accessing and engaging with HIV treatment and care. Broader social-structural reforms, such as investment in social housing [37] and drug policy reforms [7,38], should thus be pursued alongside integrated service models to maximize outcomes across the cascade of HIV care for structurally vulnerable PLHIV who use drugs.

Our findings demonstrate how the DPC’s low-barrier setting provided participants with the ability to better manage their health and wellbeing, by providing structure and enabling the development of daily routines, often structured around the DPC’s food service and medication supports. By reducing burdens associated with living in extreme poverty and managing comorbid health conditions, the DPC enabled participants to engage with a range of services (e.g. art and music therapy, group counselling) associated with improved health and wellbeing. This is particularly important as previous studies have highlighted the positive impacts of social supports and improved patient-provider relationships [39–41] on the ability of structurally vulnerable PLHIV to adhere to HIV treatment [20,22,23]. As such, the DPC represents a service model with the potential to improve retention in HIV care among PLHIV who use drugs by addressing multiple barriers to care simultaneously, and might warrant further consideration in settings implementing programmatic interventions to complement TasP.

Additionally, our work highlights the need to consider client service trajectories when examining how populations interact with service environments. This can be particularly important for participants who have delayed engagement with ancillary services. By closely exploring service interactions, our findings underscore the need for a continuum of care specific to populations’ needs, particularly when faced with evolving levels of structural vulnerability shaped by larger policies. As such, this analytical method can provide in-depth information on service usage in relation to clients’ risk environments, facilitating the tailoring of programmes to better mitigate social-structural barriers PLHIV who use drugs face in accessing and remaining retained in HIV care.

This study has several limitations. First, our findings might not be representative of the experiences of all PLHIV who use drugs accessing the DPC, despite similar demographics in comparison to the DPC’s wider client population and sampling procedures intended to promote diversity. Second, our study was conducted in a setting with universal HAART coverage and other treatment supports and, therefore, may have limited transferability to other urban settings where cost of HAART and lack of HIV-related supports may be barriers to treatment adherence. Third, women, transgender persons, and people of Indigenous ancestry were underrepresented in our sample despite efforts to oversample these populations, as only a small number access the DPC and were thus eligible for participation. Additional research is needed to fully understand their experiences with this integrated HIV care services organization. Fourth, research that tracks time in relation to how people engage with the DPC and similar services would be beneficial for understanding how length of service engagement is shaped by structural vulnerability, and may be valuable in tailoring service environments to meet the evolving need of clients.

## Conclusions

In conclusion, our study underscores the need for integrated service models to better address dynamics within the risk environment that may prevent engagement and retention in HIV care, particularly in the context of TasP. Ensuring the access to, and retention in, HIV care for structurally vulnerable PLHIV who use drugs is essential to addressing the health inequities that render particular populations at increased risk of harm.
